# Identification of a Gene-Expression-Based Surrogate of Genomic Instability during Oral Carcinogenesis

**DOI:** 10.3390/cancers14030834

**Published:** 2022-02-07

**Authors:** Eléonore Truchard, Chloé Bertolus, Pierre Martinez, Emilie Thomas, Pierre Saintigny, Jean-Philippe Foy

**Affiliations:** 1 Sorbonne Université, Department of Maxillo-Facial Surgery, AP-HP, Hôpital Pitié-Salpêtrière, 75013 Paris, France; eleonore.truchard@gmail.com (E.T.); chloe.bertolus@aphp.fr (C.B.); 2 Univ Lyon, Université Claude Bernard Lyon 1, INSERM 1052, CNRS 5286, Centre Léon Bérard, Centre de recherche en cancérologie de Lyon, 69008 Lyon, France; pierre.martinez@lyon.unicancer.fr (P.M.); pierre.saintigny@lyon.unicancer.fr (P.S.); 3 Department of Translational Medicine, Centre Léon Bérard, 69008 Lyon, France; 4 Synergie Lyon Cancer-Platform of Bioinformatics Gilles Thomas, 69008 Lyon, France; emilie.thomas@lyon.unicancer.fr; 5 Department of Medical Oncology, Centre Léon Bérard, 69008 Lyon, France

**Keywords:** oral potentially malignant disorder, oral leukoplakia, oral carcinogenesis, head and neck squamous cell carcinoma, genomic instability, gene signature, biomarker

## Abstract

**Simple Summary:**

New personalized cancer prevention strategies may decrease the mortality of oral cancer that can arise from oral potentially malignant disorders (OPMD). A major cancer hallmark is the acquisition of multiple deletions or amplifications of genomic material fragments leading to genomic instability (GI). Our goal was to identify a set of genes whose expression was associated with GI. A total of 20 genes correlated with GI were identified in two independent datasets of head and neck cancer (including oral cancer). We computed a score of those genes, referred to as the GIN score, in with each sample from multiple validation datasets. We show that the GIN score: (i) was correlated with GI, (ii) increased at different stages of oral carcinogenesis from normal mucosa to oral cancer, and (iii) was associated with malignant transformation of OPMD. The GIN score is a promising biomarker for identifying patients suffering from OPMD with high risk of oral cancer.

**Abstract:**

Background: Our goal was to identify a gene-expression-based surrogate of genomic instability (GI) associated with the transformation of oral potentially malignant disorder (OPMD) into oral squamous cell carcinoma (OSCC). Methods: GI was defined as the fraction of genome altered (FGA). Training sets included the CCLE and TCGA databases. The relevance of the enrichment score of the top correlated genes, referred to as the GIN score, was evaluated in eight independent public datasets from the GEO repository, including a cohort of patients with OPMD with available outcome. Results: A set of 20 genes correlated with FGA in head and neck SCC were identified. A significant correlation was found between the 20-gene based GIN score and FGA in 95 esophagus SCC (r = 0.59) and 501 lung SCC (r = 0.63), and in 33 OPMD/OSCC (r = 0.38). A significantly increased GIN score was observed at different stages of oral carcinogenesis (normal–dysplasia –OSCC) in five independent datasets. The GIN score was higher in 10 OPMD that transformed into oral cancer compared to 10 nontransforming OPMD (*p* = 0.0288), and was associated with oral-cancer-free survival in 86 patients with OPMD (*p* = 0.0081). Conclusions: The GIN score is a gene-expression surrogate of GI, and is associated with oral carcinogenesis and OPMD malignant transformation.

## 1. Introduction

Oral squamous cell carcinoma (OSCC) is the most common subsite of head and neck cancers [1] that is associated with a substantial morbidity and mortality, mainly due to the risk of locoregional or distant disease recurrence. Since oral squamous cell carcinoma may develop from oral potentially malignant disorder (OPMD) such as oral leukoplakia, it is the most common one, with a malignant transformation rate between 1.1% and 40.8% [2,3]. The outcome of patients suffering from OSCC could be improved by the development of robust biomarkers of the risk of OPMD malignant transformation in order to treat patients with a high risk of oral cancer using systemic agents of chemoprevention [4]. The loss of heterozygosity (LOH) at various microsatellite markers is the most robust marker of cancer risk in this setting [3,5,6,7,8,9], in line with the high genomic instability of OSCC [10,11] compared to other tumor types [12]. However, despite different retrospective and prospective studies on the predictive value of LOH for oral cancer risk, none has yet resulted in the development of a biomarker that could be considered to be the standard of care. 

Genomic instability is a major cancer hallmark [13] involving mutations and copy number alterations that play a pivotal role during tumorigenesis. As initially described in colorectal cancers [14], the accumulation of those specific genomic aberrations drives the transformation of squamous mucosa of the oral cavity during multistep tumorigenesis [15]. Because several driver genetic events may occur during this oral carcinogenesis [16], the identification of a surrogate of this genome stability could help in improving the prevention strategies in patients with OPMD. Previous studies identified gene-expression-based signatures of genomic instability in different cancer types that were associated with patients outcome [17,18,19,20].

We hypothesized that a gene-expression-based surrogate of genomic instability in HNSCC may help in identifying OPMD with a high risk of malignant transformation. Using 520 HNSCC from The Cancer Genome Atlas (TCGA) together with 32 HNSCC cancer cell lines from the Cancer Cell Line Encyclopedia (CCLE), we identified 20 genes correlated to GI, as defined by the fraction of genome altered (FGA). An enrichment score of this gene set, referred to as the genomic instability (GIN) score, was then computed in multiple datasets. The GIN score was confirmed to be associated with FGA in two independent validation datasets of squamous cell carcinoma of the esophagus and lung from TCGA. The GIN score was also associated with HNSCC subtypes, and was the highest in the classical subtype in three cohorts of patients. The GIN score increased during oral tumorigenesis and was associated with an increasing risk of oral-cancer development in patients with OPMD. 

## 2. Methods 

### 2.1. Datasets of Squamous Cell Carcinomas

#### 2.1.1. The Cancer Genome Atlas (TCGA) 

We queried the TCGA database in order to retrieve the clinical and gene-expression profiles of 520 HNSCC (HNSC-TCGA), including 421 and 97 HPV-negative and -positive tumors, respectively. Clinical data and normalized read counts generated from RNA-sequencing were downloaded using the TCGA2STAT R-package [21]. 

A similar approach was used to retrieve data from 501 lung squamous cell carcinomas (LUSC-TCGA) and 95 esophagus squamous cell carcinomas (ESCC-TCGA) from TCGA. 

Clinical data were downloaded from the cBioPortal database [22,23]. 

#### 2.1.2. Cancer Cell Line Encyclopedia

A set of 32 established cancer cell lines from upper aerodigestive tract were included in the analysis. Raw data were downloaded from ArrayExpress (E-MTAB-3610). 

#### 2.1.3. Gene Expression Omnibus

The expression profiles of 252 and 138 primary HNSCC were retrieved from the Gene Expression Omnibus database: GSE65858 [24] and GSE39366 [25], respectively. Molecular subtypes, as previously described [11,25], HPV status, and clinical information were available for tumors included in GSE39366 and GSE65858.

### 2.2. Datasets including OPMD or Oral Epithelial Dysplasia (OED)

Six independent gene-expression datasets including oral leukoplakia, the most common OPMD, or oral epithelial dysplasia (OED), as defined according to recent papers on the nomenclature and terminology of oral premalignant conditions [2,26], were downloaded from Gene Expression Omnibus and are referred to as OPMD and OED datasets. Clinical data and gene-expression profiles were retrieved in both datasets. 

The OPMD-1 dataset included 1 normal oral tissue, 15 OPMD, and 34 OSCC (GSE85195) [27]. Moreover, copy number profiles were retrieved for 10/15 OPMD and 23/34 OSCC from this dataset. Raw CGH data (Agilent Human Genotypic Designed CGH 105k Microarray) were downloaded from GEO (GSE85514) in order to perform normalization and segmentation using the rCGH R package [28]. 

RNA sequencing gene-expression profiles of 20 oral leukoplakia (OL) patients with available information regarding malignant transformation during follow-up were retrieved from GSE156206 in order to be included in the OPMD-2 dataset. During the follow-up, 10/20 OL underwent malignant transformation. The median time to malignant transformation was 17 months (IQR: 42.75 months). 

A third OPMD dataset set, OPMD-3, included whole-genome gene-expression profiles (GSE26549) of 86 OL biopsies in patients followed prospectively in a chemoprevention trial with a median follow-up of 7 years (95% CI (5.6–8.6)). In this trial, patients with OPMD were randomly assigned to treatment with 13-cisretinoic acid (13cRA) versus retinyl palmitate (RP) with or without b-carotene (BC). In 70/86 OL, we retrieved loss of heterozygosity status at different microsatellite markers 9p21 (D9S171, D9S1747), 3p14 (D3S1285), 17p13 (D17S1176), TP53, and 8p22 (D8S254) [29]. 

The OED-1 dataset included 45 normal oral tissue, 17 oral dysplasia, and 167 oral SCC (GSE30784) [30]. 

The OED-2 dataset included 30 paired normal oral tissue, dysplasia, and carcinomas (CIS or SCC) from 10 patients, with available normalized gene-expression profiles downloaded from GSE46802 [31]. The OED-3 dataset also included 33 paired oral normal, dysplasia, and SCC from 11 patients with available normalized gene-expression profiles downloaded from GSE35261 [32].

A detailed description of the different datasets, including data normalization and processing, is provided in Supplementary Table S1. 

### 2.3. Surrogates of Genomic Instability

The mutational load and fraction of genome altered (FGA), which is defined as the length of segments with log_2_ or linear CNA value larger than 0.2 divided by the length of all measured segments, were retrieved for TCGA and CCLE samples from the cBioportal database [22,23]. The FGA was also computed in OPMD and OSCC samples from GSE85515 using the CINmetrics R package [33]. 

In order to identify a gene-expression-based surrogate of genomic instability, we selected specific genes whose expression was consistently correlated associated with FGA, using a similar approach as previously described [20]. Single-sample Gene Set Enrichment Analysis tool (ssGSEA) [34,35] was run using the GSVA r package [36] in order to compute the enrichment score (ES) of those selected genes, namely, the genomic instability (GIN) score. Unlike GSEA, which analyzes differential pathways between two phenotypical groups, the ssGSEA tool allows for computing the enrichment score (ES) of a given gene set in each sample. Gene-expression values for a given sample are rank-normalized, and an ES is produced using the empirical cumulative distribution functions of the genes in the gene set and the remaining genes. 

### 2.4. Bioinformatics and Statistics

Bioinformatics and statistics were performed using Array Studio software (Omicsoft Corporation), and Bioconductor packages in the R language [19] and GraphPad Prism version 6.00 (San Diego, SA, USA). Normalization and processing of copy-number and gene-expression data are detailed for each dataset in Supplementary Table S1. 

Unpaired Mann–Whitney and Kruskal–Wallis tests were performed to compare continuous values in two or more than two groups, respectively. Paired Mann–Whitney and Friedman tests were used to compare continuous values from paired samples in two or more than two groups, respectively. The Pearson correlation coefficient (r) was estimated to measure the strength of a linear association between two continuous variables. 

Progression-free survival (PFS) was defined by time in months: from tumor biopsy to death, recurrence, or loss to follow-up (GSE39366); from tumor biopsy to a new tumor event (TCGA); from the registration date to the detection of either progression (as defined as local recurrence, new lymph node or distant metastasis, or second primary carcinoma), or death (GSE65858). In the 86 samples from the OPMD-3 dataset (GSE26549), oral cancer-free survival (OCFS) was defined as the time from the first biopsy to oral cancer or to the date of last follow-up (for censored patients). First, the association between OS, PFS, and OCFS, and the GIN score was tested using a univariate cox model. Then, the distribution of OCFS was estimated using the Kaplan–Meier method and compared with the log-rank test between groups of patients defined by the level of genomic instability (high GIN vs low GIN) according to the GIN score. The cutoff value for the GIN score to group patients into high and low GIN was determined using the Maxstat R package to identify the value that correspond to the most significant relation with OCFS. A multivariate Cox proportional hazard model, including GIN group, treatment arm (beta-carotene, 13-cis-retinoic acid or retinyl palmitate), and histological grade (hyperplasia or dysplasia) was also built to test the association of the GIN group with oral-cancer-free survival in patients with OPMD from the OPMD-3 dataset, after testing for proportional hazard assumptions. 

All statistical tests were two-sided, and P values of 0.05 or less were considered to be statistically significant. 

## 3. Results

### 3.1. Identification and Validation of Gene-Expression-Based Genomic Instability (GIN) Score

**Figure 1:**
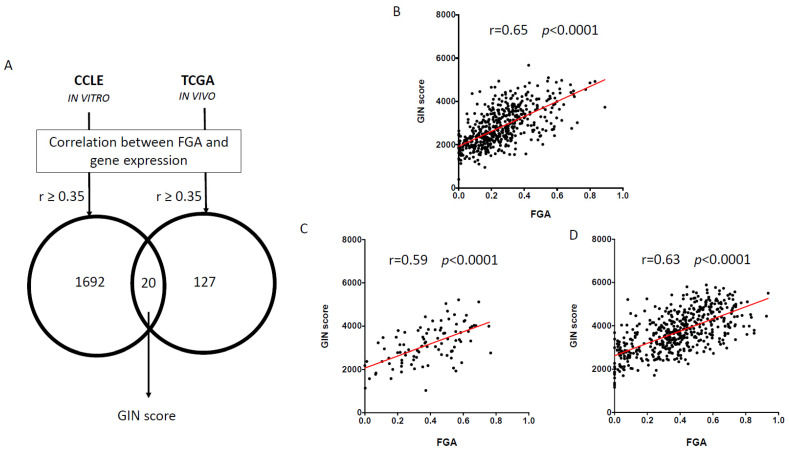
Identification and validation of GIN score. (**A**) Using Pearson’s correlation, a whole-genome ranked list of genes correlated with the fraction of genome altered (FGA) was performed in 520 HNSCC from TCGA and from 32 cancer cell lines of the upper aerodigestive tract from CCLE. Using a common threshold r ≥ 0.35 (Pearson’s coefficient correlation), we identified a total of 20 genes overlapping between the two datasets. From this set of 20 genes, we computed the GIN score in (**B**) 520 HNSCC, (**C**) 95 esophagus SCC and (**D**) 501 lung SCC, and from TCGA using the ssGSEA tool in order to test the correlation between this score and FGA. r Pearson’s coefficient is shown.

Using a similar approach as previously described [20], we tested the correlation between whole-genome expression and the fraction of genome altered (FGA), a measure of genomic instability, in 520 HNSCC from TCGA and in 32 cancer cell lines of the upper aerodigestive tract from CCLE. All genes were ranked according to Pearson’s coefficient of correlation in the TCGA-HNSC and CCLE datasets. Different thresholds of correlation (≥0.2, ≥0.3, ≥0.35, ≥0.4) were tested in order to select a relevant number of genes (~10 to 50) to be included into a gene signature of genomic instability (Supplementary Table S2). Using a threshold of 0.35, a total of 20 overlapping genes between these two datasets was identified (Figure 1A) and selected for further analysis (Table 1). There were no overlapping genes between those genes and the CIN70 signature that had been established and validated as a surrogate of chromosomal instability across different cancer types. Pathway enrichment of this set of 20 genes was analyzed using the EnrichR tool [37,38,39]. The two most enriched terms from the Reactome 2016 library were ‘chromatin organization Homo sapiens R-HSA-4839726′ and ‘chromatin modifying enzymes Homo sapiens R-HSA-3247509′ (Q-value = 0.0443), related to 3 genes (MTA1, SMYD3, CDK4) from our gene set.

Using the ssGSEA tool, we computed the enrichment score of those 20 genes, referred to as the GIN score, in the 520 HNSCC sample from TCGA-HNSC, and in the 501 lung squamous cell carcinoma (LUSC) and 95 esophagus squamous cell carcinoma (ESCC) from the TCGA-LUSC and TCGA-ESCC datasets, respectively. In order to validate this score as a surrogate of genomic instability, we tested the correlation between GIN score and FGA in both datasets. Significant correlation was found between FGA and GIN score in TCGA-HNSC (r = 0.65, *p* < 0.0001, Figure 1B), and in TCGA-ESCC (Figure 1C, r = 0.59, *p* < 0.0001) and TCGA-LUSC (r = 0.63, *p* < 0.0001, Figure 1D). Conversely, the GIN score was not consistently correlated with the mutation count in samples from TCGA-HNSC (r = 0.19, *p* = 0.0012), TCGA-LUSC (r = 0.01, *p* = 0.8958), and TCGA-ESCC (r = –0.09, *p* = 0.4018). 

Lastly, GIN score and FGA were computed in 10 OPMD and 23 OSCC from the OPMD-1 dataset. Significant positive correlation was also found between GIN score and FGA during oral carcinogenesis (r = 0.38, *p* = 0.0292). 

### 3.2. GIN Score Is Associated with the Molecular Classification of HNSCC 

**Figure 2:**
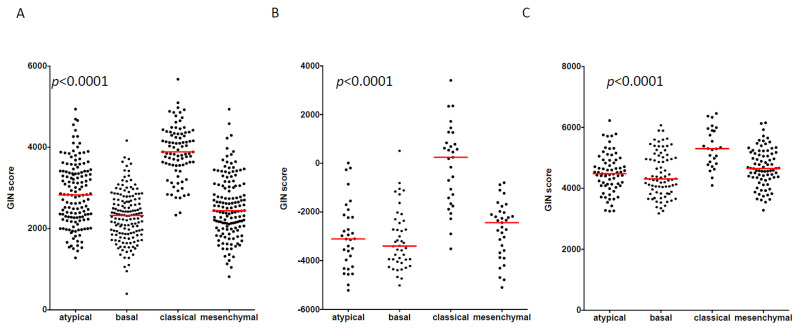
Association of GIN score with molecular classification of HNSCC. GIN score was computed in 520, 138, and 253 HNSCC from TCGA (**A**), GSE39366 (**B**), and GSE65558 (**C**) respectively. In all 3 datasets, GIN score was compared among the four previously described molecular subtypes (At: atypical; Ba: basal; Cl: classical; Me: mesenchymal) using a Kruskall–Wallis test. *p*-value is shown.

In large genomic profiling studies of HNSCC, four distinct molecular subtypes are consistently reported: atypical, basal, classical, and mesenchymal [11,25]. The classical subtype of HNSCC is recognized to harbor a high level of genomic alterations [14,15] compared to others. The GIN score was computed using the ssGSEA tool in 520, 138 and 253 HNSCC from TCGA-HNSC, GSE39366, and GSE65858, respectively. The GIN score was statistically different across molecular subtypes in both datasets (*p* < 0.0001), and consistently higher in classical compared to atypical, basal, and mesenchymal HNSCC (Figure 2A–C). No significant difference was found between HPV-negative and -positive HNSCC in both datasets (Supplementary Figure S1). 

We tested the association of the GIN score with the survival of patients with HNSCC from TCGA-HNSC, GSE39366, and GSE65858. No significant association was found between GIN score and survival using a univariate Cox model in patients from TCGA (OS: *p* = 0.078; PFS: *p* = 0.463), GSE39366 (PFS: *p* = 0.493) and GSE65858 (OS: *p* = 0.393; PFS: *p* = 0.398), and using Kaplan–Meier curves and a log-rank test (Supplementary Figures S2–S4). 

### 3.3. GIN Score Increased from Dysplasia to OSCC during Oral Carcinogenesis

In order to assess the dynamics of the GIN score at different stages of oral carcinogenesis, we computed the GIN score in five independent datasets: (i) oral normal tissue, (ii) oral epithelial dysplasia (OED) or oral potentially malignant disorders (OPMD), and (iii) oral carcinomas.

**Figure 3:**
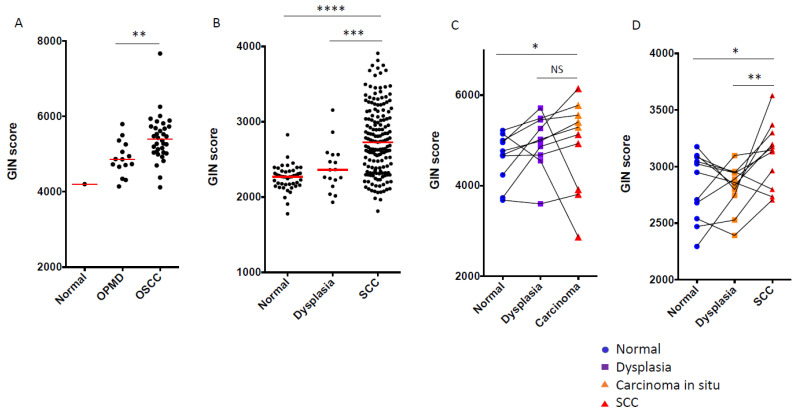
Dynamics of GIN score during oral carcinogenesis. In order to assess its dynamics during oral carcinogenesis, the GIN score was computed in: (**A**) 1 normal oral mucosa, 15 OPMD, and 34 OSCC from the OPMD-1 dataset; (**B**) 45 normal oral mucosa, 17 oral epithelial dysplasia, and 167 OSCC from the OED-1 dataset; (**C**,**D**) 30 and 33 paired normal mucosa, epithelial dysplasia and carcinoma (CIS or SCC) samples of the oral cavity from 10 and 11 patients included in the (**C**) OED-2and (**D**) OED-3 datasets, respectively. GIN score was compared between paired samples (connected with black lines in panels **C**,**D**) using a paired Mann–Whitney Test and a Friedman test in two and more than two groups, respectively. Unpaired Mann–Whitney and Kruskall–Wallis tests were used to compare samples in (**A**) two groups and (**B**) more than two groups, respectively. * *p* < 0.05, ** *p* < 0.01, *** *p* < 0.001, **** *p* < 0.0001. NS: not significant.

First, the GIN score was computed in the OPMD-1 dataset including 1 normal oral mucosa, 15 oral leukoplakia (OL), and 34 OSCC (Figure 3A), and was higher in OSCC compared to OPMD (Mann–Whitney test, *p* = 0.0016). 

Moreover, in the OED-1 dataset including 45 normal tissue, 17 oral dysplasia, and 167 OSCC, the GIN score was also significantly increased from normal to dysplasia and OSCC (Figure 3B, Kruskal–Wallis Test, *p* < 0.0001).

The GIN score was then computed in 30 paired oral normal mucosa, epithelial dysplasia, and CIS/OSCC from 10 patients included in the OED-2 dataset (Figure 3C). The GIN score was statistically different among normal, dysplasia, and SCC samples (Friedman test, *p* = 0.0303). The GIN score was increased from dysplasia to OSCC in 8/10 patients, but did not reach statistical significance (Mann–Whitney test, *p* = 0.4316). In an independent dataset (OED-3) composed of 33 paired oral normal mucosa, epithelial dysplasia, and OSCC from 11 patients (Figure 3D), the GIN score was also statistically different among normal, dysplasia, and SCC samples (Friedman test, *p* = 0.0273), with a significant increase from dysplasia to OSCC (Mann–Whitney test, *p* = 0.0098). 

Lastly, in the OPMD-3 dataset, we found an increased GIN score from OPMD with no histological change to OPMD with hyperplasia to OPMD with mild, moderate, or severe dysplasia *(p* = 0.0276, Supplementary Figure S5). 

Overall, the GIN score was statistically different between samples at different histological steps of oral carcinogenesis. While the difference in GIN score between normal and dysplasia was not consistent across samples and datasets, an increased GIN score was more pronounced from dysplasia or OPMD to OSCC, suggesting the potential relevance of this score to predict oral-cancer development. 

### 3.4. GIN Score Is Associated with Oral-Cancer Development in Patients with Oral Leukoplakia

**Figure 4:**
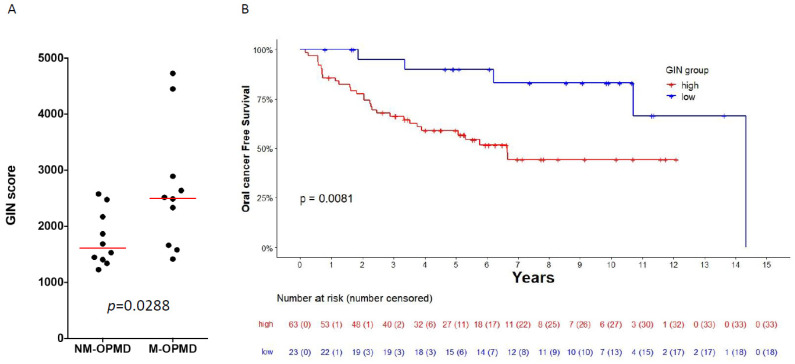
Association of GIN score with OPMD malignant transformation. (**A**) GIN score was computed in 20 OPMD from the OPMD-2 dataset, with a long-term follow-up regarding malignant transformation, and was compared between OPMD with malignant transformation (M-OPMD) and 10 OPMD without malignant transformation (NM-OPMD). (**B**) GIN score was also computed in 86 OPMD from the OPMD-3 dataset. Samples were grouped into low and high GIN according to optimal cutoff for GIN score using the Maxstat r package in order to evaluate the association of the GIN score with oral cancer-free survival (OCFS), using Kaplan–Meier curves. Survival distribution compared between high and low GIN groups using a log-rank test.

Because our data suggest that the GIN score could be associated with oral-cancer development, we computed the GIN score in 10 oral leukoplakia that had transformed into OSCC during follow-up and 10 oral leukoplakia without malignant transformation (OPMD-2 dataset). The score was significantly higher in malignant transforming OPMD (M-OPMD) compared to that in nonmalignant transforming (NM-OPMD) (Figure 4A, Mann–Whitney test, *p* = 0.0288). 

The score was also computed in 86 OPMD from the OPMD-3 dataset in order to evaluate the association of the GIN score with oral-cancer-free survival (OCFS) using Kaplan–Meier curves (Figure 4B). Using a univariate Cox model, an increased GIN score was associated with improved OCFS (*p* = 0.0118). The enrichment score of the pan-cancer chromosomal instability signature (CIN70), as previously described, was also computed in the 86 OL. No significant association was found between CIN 70 score and OCFS in this dataset’s (*p* = 0.25) univariate Cox model. Then, in order to group patients into low and high levels of genomic instability (GI) according to GIN score, we used the Maxstat R package [40] in order to identify the optimal GIN score threshold (score = 82.88) that corresponded to the most significant relation with oral-cancer-free survival. Patients suffering from OPMD with a high GIN score (>threshold = 82.88) had shorter oral-cancer-free survival compared to OPMD with a low GIN score (*p* = 0.0081). Using a multivariate Cox model including histological grade (hyperplasia vs dysplasia) and treatment arm, patients suffering from OPMD with a high GIN score had shorter oral-cancer-free survival (HR = 3.55, IC95 (1.23;10.28), *p* = 0.0193, Supplementary Table S3). 

Lastly, using our previously published molecular classification of OPMD, we found that GIN score was significantly higher in the classical subtype compared to that in the immunological subtype (*p* = 0.0003, Supplementary Figure S5) while there was no overlapping gene between the GIN signature and our previous 400-gene classifier. No significant association was found between the LOH status at different microsatellite markers and the GIN score (Supplementary Figure S6). 

## 4. Discussion

Oral carcinogenesis is characterized by the accumulation of key genetic events [15,16] leading to overall genomic instability. At premalignant steps, the loss of heterozygosity, which may occur at different microsatellite markers, is the most robust marker of this instability, which is associated with OPMD malignant transformation [5,6,7,8,41,42]. Over the single biomarker approach, we aimed to capture an overall genomic instability phenotype in OPMD. Because genomic instability is an intrinsic property of cancer cells, we first identified a set of 20 genes whose expression was correlated with genomic instability, as defined by the fraction of genome altered, in two independent datasets of HNSCC from TCGA and the CCLE. Using the ssGSEA tool, we computed a genomic instability (GIN) score corresponding to the enrichment score of this set of genes in independent datasets in order to validate the correlation between this score and the FGA. Lastly, we found that the GIN score was associated with oral carcinogenesis in independent datasets of OPMD. 

The gene-expression-based surrogate of genomic instability had been proposed in different cancer types, including breast and colorectal cancers [17,18,19,20,43]. The CIN70 signature [20] is the most robust gene-expression marker of chromosomal instability that is associated with prognosis [44,45]. In patients suffering from HNSCC, a significant association was recently observed between this signature and survival [46]. However, to the best of our knowledge, there is no gene signature associated with genomic instability at premalignant steps of head and neck tumorigenesis. We used a similar approach to identify the specific genes whose expression was correlated with genomic instability, as defined by the fraction of genome altered. In our study, we showed significant correlation between GIN score and FGA in independent datasets, while correlation between GIN score and mutation count was not significant and consistent across datasets. Thus, the GIN score would allow for capturing a GI related to copy-number variations rather than to the mutational load. Pathway enrichment analysis of our set of 20 genes showed that the most enriched terms were related to chromatin organization involving 3/20 genes (MTA1, SMYD3, CDK4), suggesting that we identified a signature of GI related to chromosomal rearrangements. 

We observed an increased GIN score at different stages of oral tumorigenesis (normal—dysplasia/OPMD/OSCC) in independent datasets. Moreover, we observed significant association with the GIN score with oral-cancer-free survival (OCFS) in 86 patients with OPMD, while no association was found between CIN70 score and OCFS. There was no overlapping gene between the GIN and the CIN70 signatures, suggesting that the GIN score could add relevance to the prognostic value of the CIN70 signature, as previously shown in various cancer types. While the CIN70 signature was associated with survival of patients suffering from HNSCC [46], our approach allowed for us to identify specific genes that were associated with FGA during oral carcinogenesis and with the malignant transformation of OPMD. 

We identified two gene-expression-based molecular subtypes of OPMD using a 400-gene classifier. There were no overlapping genes between GIN signature and this classifier, and we observed that the GIN score was significantly higher in classical OPMD compared to that in immunological OPMD. Moreover, no significant association was found between LOH status, available in 70/86 OPMD from the OPMD-5 dataset, and GIN score, suggesting the need for combining our GI signature with other biomarkers together with our previous molecular classification of OPMD. 

## 5. Conclusions

The GIN score is a gene-expression-based surrogate of genomic instability associated with oral carcinogenesis and OPMD malignant transformation. Large prospective cohorts of OPMD are needed to validate our results and refine the optimal cutoff for the GIN score for the assessment of oral-cancer risk. Further integration of this score with other potential biomarkers associated with OPMD malignant transformation is required, which may pave the road to innovative chemoprevention strategies.

## Figures and Tables

**Table 1:** List of genes included in the GIN score.

Symbol	ID	Chr. Site	Description	Cor CCLE	Cor TCGA
**APMAP**	57136	20p11.2	Adipocyte plasma membrane associated protein	0.562	0.35
**CDK4**	1019	12q14	Cyclin-dependent kinase 4	0.364	0.36
**CECR5**	27440	-	Cat eye syndrome chromosome region, candidate 5	0.383	0.359
**CNNM1**	26507	10q24.2	Cyclin and CBS domain divalent metal cation transport mediator 1	0.388	0.353
**DLX6**	1750	7q22	Distal-less homeobox 6	0.373	0.357
**FLVCR1**	28982	1q32.3	Feline leukemia virus subgroup C cellular receptor 1	0.378	0.432
**GLI2**	2736	2q14	GLI family zinc finger 2	0.38	0.364
**GTPBP3**	84705	19p13.11	GTP binding protein 3 (mitochondrial)	0.355	0.372
**MTA1**	9112	14q32.3	Metastasis-associated 1	0.367	0.385
**PEX5**	5830	12p13.31	Peroxisomal biogenesis factor 5	0.376	0.363
**PMS2P1**	5379	7q22.1	PMS1 homolog 2, mismatch repair system component pseudogene 1	0.426	0.406
**PNCK**	139728	Xq28	Pregnancy upregulated nonubiquitous CaM kinase	0.396	0.458
**SLC30A3**	7781	2p23.3	Solute carrier family 30 (zinc transporter), member 3	0.449	0.351
**SMYD3**	64754	1q44	SET and MYND domain containing 3	0.47	0.364
**TFB2M**	64216	1q44	Transcription factor B2, mitochondrial	0.382	0.357
**TMEM161A**	54929	19p13.11	Transmembrane protein 161A	0.357	0.369
**TMEM97**	27346	17q11.2	Transmembrane protein 97	0.478	0.371
**USP39**	10713	2p11.2	Ubiquitin specific peptidase 39	0.409	0.392
**VMA21**	203547	Xq28	VMA21 vacuolar H+-ATPase homolog (*S. cerevisiae*)	0.373	0.371
**ZNF74**	7625	22q11.21	Zinc finger protein 74	0.423	0.366

## Data Availability

All data are available from public repositories The Cancer Genome Atlas, The Cancer Cell Line Encyclopedia, Gene Expression Omnibus, and ArreyExpress.

## Supplementary Materials

The following supporting information can be downloaded at: https://www.mdpi.com/article/10.3390/cancers14030834/s1, Table S1: Description of the 12 datasets used in our study, Table S2: Number of selected genes according to the threshold of correlation, Table S3: Multivariate oral cancer-free survival analysis of patients with OPMD from the OPMD-3 dataset (GSE26549). Figure S1: Association of the GIN score and HPV status in HNSCC from TCGA (A), GSE39366 (B) and GSE65858 (C). Figure S2: Association of the GIN score and survival in HNSCC from TCGA (Samples were grouped into “low” and “high” GI according to the median of the GIN score, in order to evaluate the association of the GIN score with progression-free survival (PFS) (A) and overall survival (OS) (B) using Kaplan-Meier curves. Figure S3: Association of the GIN score and survival in HNSCC from GSE39366 Samples were grouped into “low” and “high” GI according to the median of the GIN score, in order to evaluate the association of the GIN score with progression-free survival (PFS) using Kaplan-Meier. Figure S4: Association of the GIN score and survival in HNSCC from GSE65558. Samples were grouped into “low” and “high” GI according to the median of the GIN score, in order to evaluate the association of the GIN score with progression-free survival (PFS) (A) and overall survival (OS) (B) using Kaplan-Meier. Figure S5: Association of the GIN score with histological step of oral carcinogenesis (normal-hyperplasia-dysplasia) (A) and the molecular classification of OPMD into the classical and immunological subtypes (B). The GIN score was compared between histological steps and between classical and immunological OPMD using a Kruskall-Wallis Test and Mann-Whitney Test respectively. Figure S6: Association of the GIN score with LOH status. The GIN score was compared between LOH+ and LOH− at different microsatellite markers: 9p21 (D9S171, D9S1747), 3p14 (D3S1285), 17p13 (D17S1176), TP53, 8p22 (D8S254), and for any LOH.
