# Supplementary material for: Identification of a Gene-Expression-Based Surrogate of Genomic Instability during Oral Carcinogenesis
Source: Cancers (Basel). 2022 Feb 7;14(3):834. doi: 10.3390/cancers14030834 (PMC8834046; doi:10.3390/cancers14030834)
Supplement: Supplementary file 1 [file cancers-14-00834-s001.zip › cancers-1559245-supplementary.pdf]

Table S1. Description of the 12 datasets used in our study.

Datasets *ref GEO*	Sample type	Total (n=)	Platform	Normalization	Ref
**OED-1** *GSE30784*	Oral mucosa	45 normal<br>17 dysplasia<br>167 OSCC	Affymetrix Human Genome U133 Plus 2.0	Raw data (CEL files) were processed using quantile normalization and the robust multi-array average (RMA) algorithm, in addition to the custom CDF version 18 from brainarray. Data was then $\log_2$ transformed.	[1]
**OED-2** *GSE46802*	Oral mucosa	30 paired samples from 10 patients<br>- 10 normal<br>- 10 dysplasia<br>- 10 cancer (in situ or SCC)	Agilent-014850 whole human genome microarray 4×44	Normalized data as described in the original publication were directly extracted from Gene Expression Omnibus data portal. All probe set expression values for a given gene were median-collapsed into a single expression value.	[2]
**OED-3** ***GSE35261***	Oral mucosa	30 paired samples from 11 patients<br>-11 normal<br>- 11 dysplasia<br>-11 SCC	Operon Human 37.6K V4.0.1 Oparray	Normalized data as described in the original publication were directly extracted from Gene Expression Omnibus data portal. All probe set expression values for a given gene were median-collapsed into a single expression value.	[3]
**OPMD-1**	Oral Mucosa	62 samples (including OL and OSCC)			
- GSE85195	Gene expression	33/62 samples with available CNA and GE profiles between GSE85195 and GSE85514:<br>- 10 OL<br>- 23 OSCC	Agilent-014850 whole human genome microarray 4×44	Normalized data as described in the original publication were directly extracted from Gene Expression Omnibus data portal. All probe set expression values for a given gene were median-collapsed into a single expression value.	[4]
- GSE85514	Copy number		Agilent-014850 whole human genome microarray 4×44	Raw data were downloaded. Normalization and segmentation was performed using the rCGH r package	
**OPMD-2** *GSE156208*	OL	- 10 OL with malignant transfotmation<br>- 10 OL without malignant transformation	Illumina NextSeq 500 RNA sequencing	RNA-seq read counts were downloaded from GEO et log-2 transformed.	[5]
**OPMD-3** *GSE26549*	OL	86	Affymetrix Human 1.0 ST	Raw data (CEL files) were processed using quantile normalization and the robust multi-array average (RMA) algorithm, in addition to the custom CDF version 18 from brainarray. Data was then $\log_2$ transformed.	[6]
**GSE39366**	Primary HNSCC	138	Agilent-UNC-custom-4X44K	Normalized data as described in the original publication were directly extracted from Gene Expression Omnibus data portal. All probe set expression values for a given gene were median-collapsed into a single expression value.	[7]
**GSE65858**	Primary HNSCC	252	Illumina HumanHT-12 V4.0	Normalized data as described in the original publication were directly extracted from Gene Expression Omnibus data portal. All probe set expression values for a given gene were median-collapsed into a single expression value.	[8]
**CCLE** *E-MTAB-3610*	Cell lines from UADT	33	Affymetrix Human Genome U133 Plus 2.0	Raw data (CEL files) were processed using quantile normalization and the robust multi-array average (RMA) algorithm, in addition to	[9,10]

				the custom CDF version 18 from brainarray. Data was then log_2 transformed.	
**TCGA-HNSC**	HNSCC	521	Illumina HiSeq 2000 RNA sequencing	RNA seq version 2 (level III). Using the TCGA2STAT R package, we downloaded data that were normalized using MapSlice to do the alignment and RSEM to perform the quantitation.	[11]
**TCGA-LUSC**	LUSC	501	Illumina HiSeq 2000 RNA sequencing	RNA seq version 2 (level III). Using the TCGA2STAT R package, we downloaded data that were normalized using MapSlice to do the alignment and RSEM to perform the quantitation.	[11]
**TCGA-ESCC**	ESCC	96	Illumina HiSeq 2000 RNA sequencing	RNA seq version 2 (level III). Using the TCGA2STAT R package, we downloaded data that were normalized using MapSlice to do the alignment and RSEM to perform the quantitation.	[11]

GEO: gene expression omnibus; GE: gene expression; CNA: copy number alterations; HNSCC: Head and Neck Squamous Cell Carcinomas; IC50: half maximal inhibitory concentration; LUSC: lung squamous cell carcinoma; ESCC: esophagus squamous cell carcinoma; OL: oral leukoplakia; UADT: upper aerodigestive tract; OPMD: oral potentially malignant disorder; OED: oral epithelial dyplasia; TCGA: The Cancer Genome Atlas; CCLE: cancer cell line encyclopedia.

Table S2. Number of selected genes according to the threshold of correlation.

Threshold_r	Nb genes_CCLE	Nb genes_TCGA	Overlap
≥0.2	4773	2161	734
≥0.3	2543	451	95
≥0.35	1712	147	20
≥0.4	1044	31	1

Table S3. Multivariate oral cancer-free survival analysis of patients with OPMD from the OPMD-3 dataset (GSE26549).

	HR	CI95%	P-value
**GIN group**			
High vs low	3.55	[1.23;10.28]	0.0193
**histology**	0.77	[0.39;1.54]	0.4635
**Treatment**	0.98	[0.70;1.38]	0.9053

A multivariate cox model, including the GIN group, the treatment arm (beta-carotene, 13-cis-retinoic acid or retinyl palmitate) and the histological grade (hyperplasia or dysplasia), was performed to test the association of the GIN group with oral cancer-free survival in 86 patients with OPMD, followed prospectively in a chemoprevention trial. Two groups of patients were defined by the level of genomic instability (high GI vs low GI) according to the GIN score. The cutoff value for the GIN score (=82.88) to group patients into high (>cutoff) and low (≤ cutoff) GI was determined using the Maxstat r package to identify the value that correspond to the most significant relation with OCFS. HR: hazard ratio; CI: confidence interval.

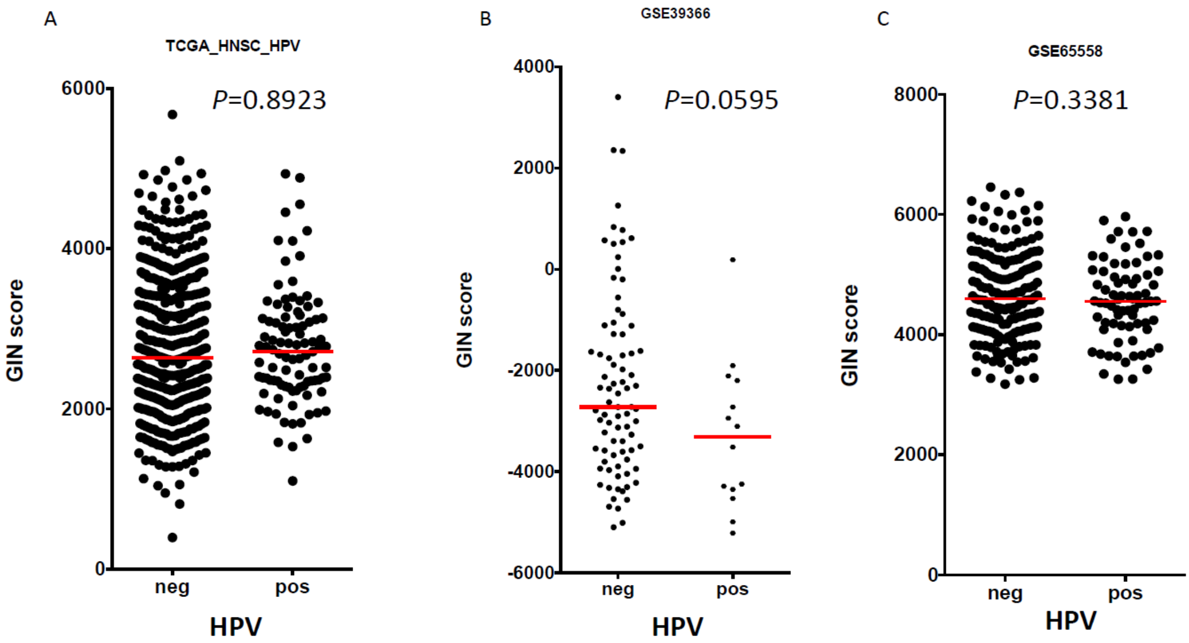

Figure S1. Association of the GIN score and HPV status in HNSCC from TCGA (**A**), GSE39366 (**B**) and GSE65858 (**C**).

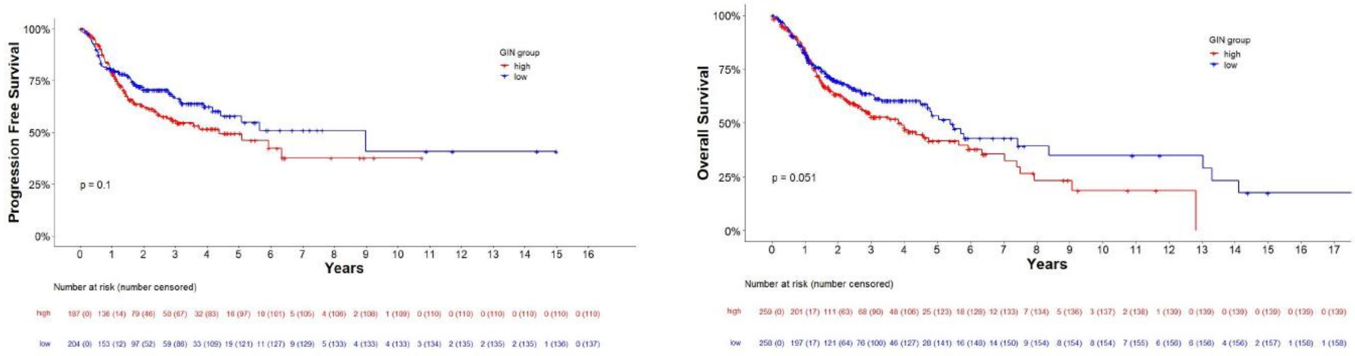

Figure S2. Association of the GIN score and survival in HNSCC from TCGA (Samples were grouped into “low” and “high” GI according to the median of the GIN score, in order to evaluate the association of the GIN score with progression-free survival (PFS) (**A**) and overall survival (OS) (**B**) using Kaplan-Meier curves.

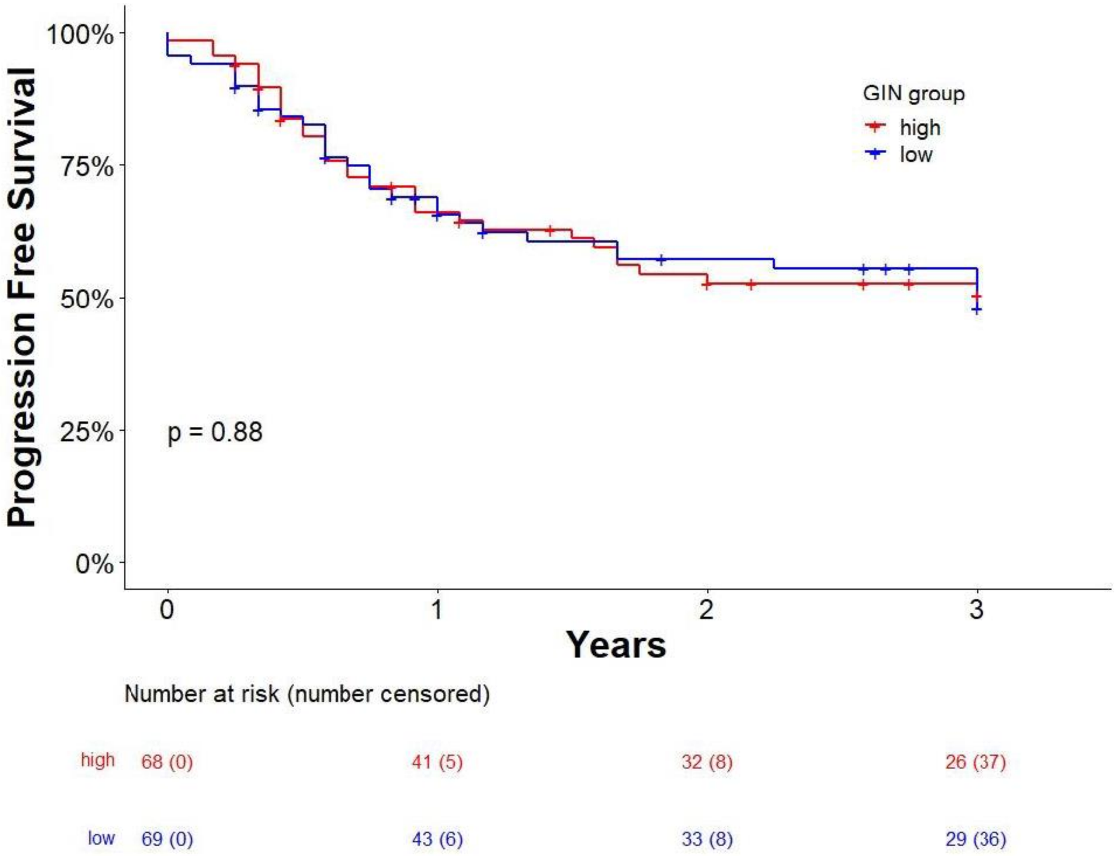

Figure S3. Association of the GIN score and survival in HNSCC from GSE39366 Samples were grouped into "low" and "high" GI according to the median of the GIN score, in order to evaluate the association of the GIN score with progression-free survival (PFS) using Kaplan-Meier.

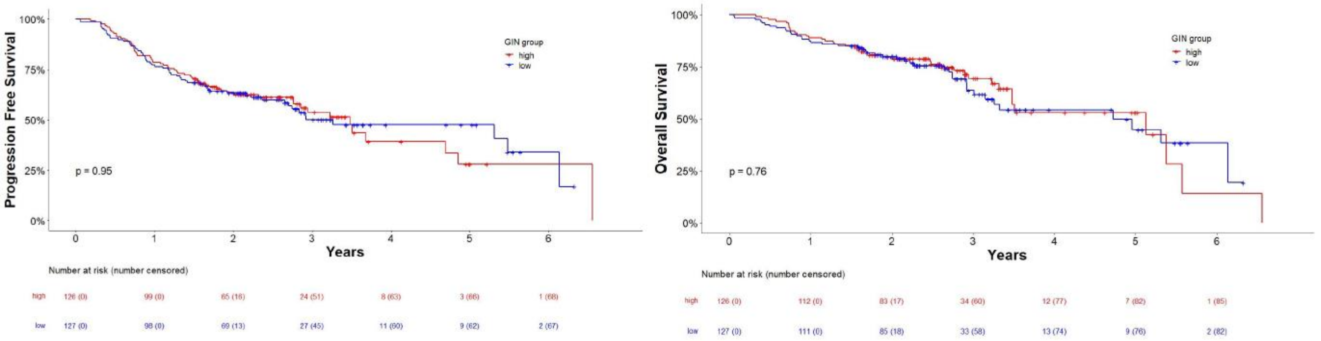

Figure S4. Association of the GIN score and survival in HNSCC from GSE65558. Samples were grouped into "low" and "high" GI according to the median of the GIN score, in order to evaluate the association of the GIN score with progression-free survival (PFS) (**A**) and overall survival (OS) (**B**) using Kaplan-Meier.

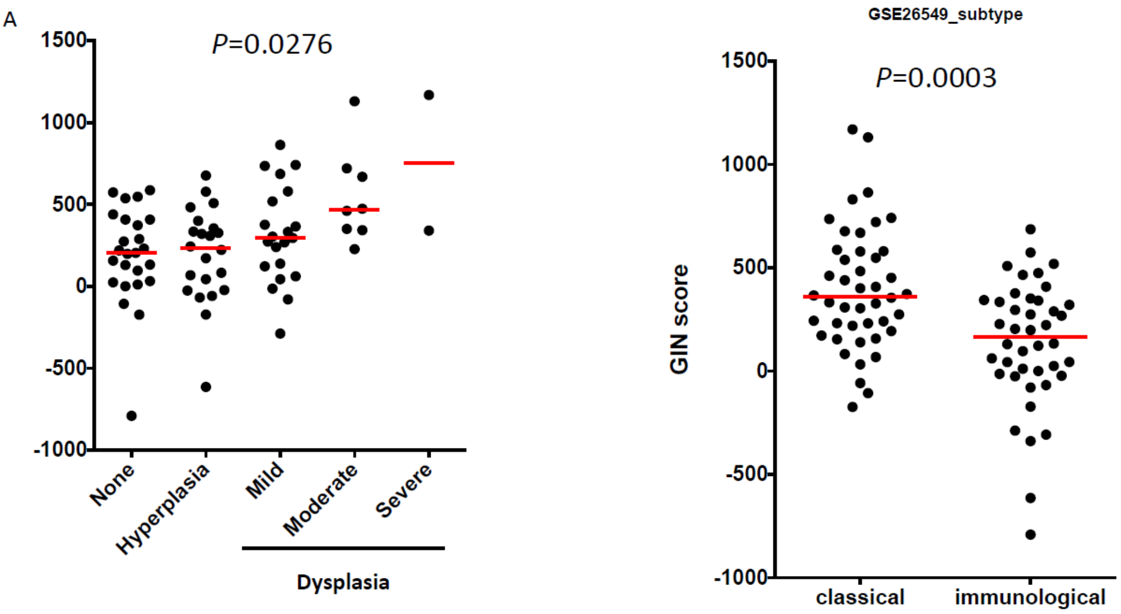

Figure S5. Association of the GIN score with histological step of oral carcinogenesis (normal-hyperplasia-dysplasia) (**A**) and the molecular classification of OPMD into the classical and immunological subtypes (**B**). The GIN score was compared between histological steps and between classical and immunological OPMD using a Kruskall-Wallis Test and Mann-Whitney Test respectively.

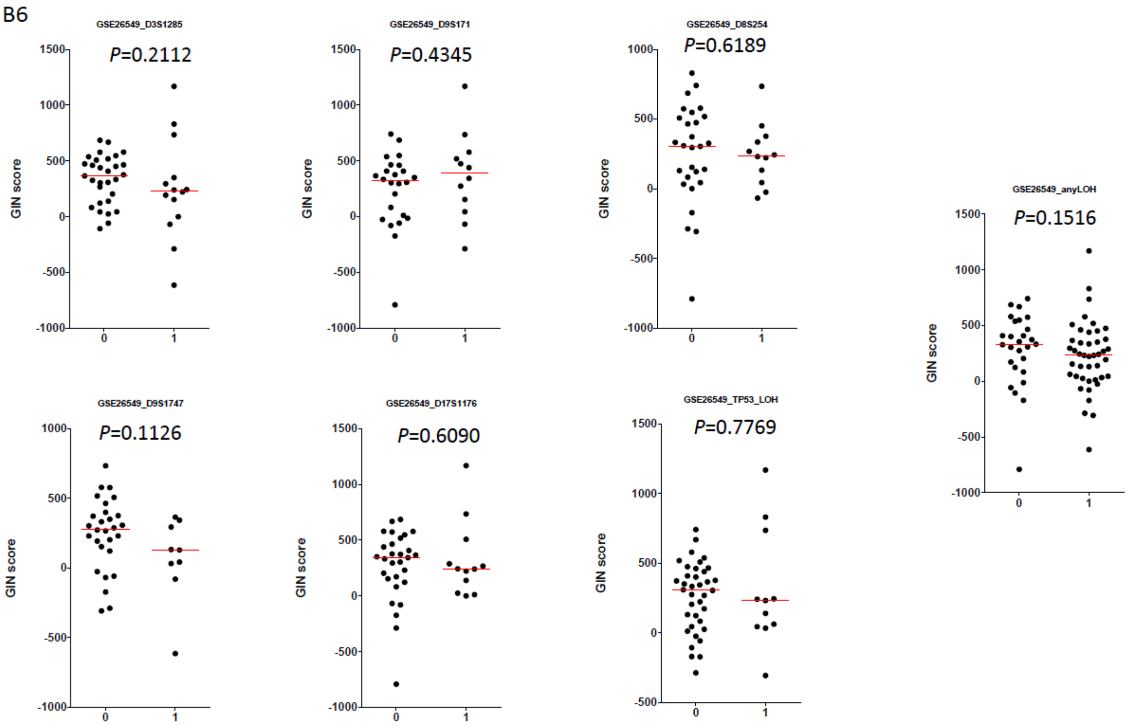

Figure S6. Association of the GIN score with LOH status. The GIN score was compared between LOH+ and LOH- at different microsatellite markers: 9p21 (D9S171, D9S1747), 3p14 (D3S1285), 17p13 (D17S1176), TP53, 8p22 (D8S254), and for any LOH.
